# Pharmakotherapie der Alkoholentwöhnung: Update und neue Entwicklungen

**DOI:** 10.1007/s00115-020-00954-5

**Published:** 2020-07-21

**Authors:** Michael Soyka, Susanne Rösner

**Affiliations:** 1 Medical Park Chiemseeblick, Rasthausstraße 25, 83233 Bernau/Felden, Deutschland; 2 Psychiatrische Universitätsklinik, LMU München, München, Deutschland; 3 Forel Klinik, Ellikon an der Thur, Schweiz

**Keywords:** Alkoholgebrauchsstörungen, Anti-Craving-Medikamente, Rückfall, Acamprosat, Naltrexon, Alcohol use disorders, Anti-Craving-Drugs, Relapse, Acamprosate, Naltrexone

## Abstract

Bislang sind nur wenige Medikamente zur pharmakologischen Rückfallprophylaxe der Alkoholabhängigkeit zugelassen. Neben dem in Deutschland nicht mehr vertriebenen Disulfiram sind es die Opioidantagonisten Naltrexon und Nalmefen sowie das vermutlich über glutamaterge Neurone wirkende Acamprosat. Baclofen und γ‑Hydroxybutyrat (GHB) sind in einzelnen Ländern zugelassen. Wirkstoffe wie z. B. Vareniclin, Gabapentin und Topiramat können für die Rückfallprophylaxe der Alkoholabhängigkeit von Interesse sein, jedoch ist bislang keine Zulassung erfolgt. Vor dem Hintergrund der zur Revision anstehenden S3-Leitlinie zur Diagnose und Behandlung alkoholbezogener Störungen wird der heutige Kenntnisstand zur Pharmakotherapie der Alkoholabhängigkeit dargestellt.

## Abstract (en)

So far few medications are approved for prophylactic treatment of alcohol dependence relapse. Apart from disulfiram, which is no longer marketed in Germany, the opioid antagonists naltrexone, nalmefene and the putative glutamate antagonist acamprosate are approved. In some other countries, baclofen and gamma-hydroxybutyrate (GHB) are licensed. Possible other drugs of interest for prophylaxis of alcohol dependence relapse are vareniclin, gabapentin, and topiramate, but so far none of them have received approval. In the light of the currently running revision of the German guidelines for the diagnosis and treatment of alcohol related disorders, an update on the pharmacotherapy of alcohol dependence is presented.

## Einleitung

Während in der ICD-10 [22] schädlicher Gebrauch und Abhängigkeit von Alkohol unterschieden werden, folgte das DSM‑5 ([8], Übersicht in [53]) einem dimensionalen Ansatz und definiert Alkoholkonsumstörungen anhand von 11 Kriterien, von denen mindestens 2 bis 3 für eine leichte Störung definiert sein müssen. Die 2022 in Kraft tretende ICD-11 wird an der Unterscheidung schädlicher Gebrauch und Abhängigkeit festhalten. Alkoholgebrauchsstörungen sind häufig. In vielen epidemiologischen Untersuchungen werden Prävalenzraten von 6 bis 7 % mitgeteilt [34, 53]. Etwa 2 bis 3 % der Erwachsenenbevölkerung sind alkoholabhängig. Zu den zahlreichen körperlichen und neurologischen Folgeschäden gehören Lebererkrankungen, eine erhöhte Krebsrate, ein signifikantes Unfall- und Suizidrisiko, aber auch das Risiko für Gewalttaten, zahlreiche psychiatrische Folgestörungen sowie soziale Probleme [81]. Die Prognose ist bei hoher Mortalität/Morbidität in vielen Fällen immer noch ungünstig [24, 61]. Die verfügbaren Ansätze zur Therapie von Alkoholkonsumstörungen umfassen ein breites und umfangreiches Spektrum [59, 63, 64, 71]. Trotz erwiesener Effizienz etablierter Therapien sind Konsum- und Rückfallereignisse häufig.

Die 2016 publizierte S3-Leitlinie zu Screening, Diagnose und Behandlung alkoholbezogener Störungen [59] befindet sich derzeit in Revision. Als übergeordnete Therapieziele wurden in der S3-Leitlinie aus 2016 neben abstinenzorientierten Therapien auch Harm-reduction-Strategien (Verminderung der Trinkmenge) als mögliche Behandlungsziele definiert.

Als wirksame psychosoziale und psychotherapeutische Behandlungsansätze in der Entwöhnungsbehandlung der Alkoholabhängigkeit sind Interventionskomponenten wie z. B. motivationale Interventionsformen, Verhaltenstherapie und kognitive Verhaltenstherapie wie Kontingenzmanagement, Angehörigenarbeit und Paartherapie mit hohem Empfehlungsgrad genannt. Mitunter wird bei insgesamt moderaten Effekten eine leichte Überlegenheit der kognitiven Verhaltenstherapien gegenüber anderen spezifischen Therapien vermutet, die jedoch in einer aktuellen Metaanalyse [58] nicht bestätigt wurde. Darüber hinaus finden in der S3-Leitlinie die psychotherapeutische Kurzzeittherapie, kognitives Training sowie andere Psychotherapieformen Erwähnung.

Als Medikamente sind Acamprosat, Naltrexon und Disulfiram empfohlen. Für Acamprosat und Naltrexon war bei einer sehr guten Evidenzbasierung (Level 1a) allerdings nur der Empfehlungsgrad B („sollte gegeben werden“) ausgesprochen worden. Disulfiram erhielt bei Evidenzbasierung 1b den Empfehlungsgrad 0. Die Datenlage zur Empfehlung von Nalmefen war bei Erstellung der Leitlinien noch nicht ausreichend. Tab. 1 gibt einen Überblick über die zur Pharmakotherapie der Alkoholabhängigkeit eingesetzten Substanzen.

**Table 1:** 

Medikamente	Dosis	Wirkmechanismus	Andere Indikationen
Acamprosat	1998 mg/Tag	Unklar, NMDA-Rezeptor-Agonist, Modulator hyperaktiver glutamaterger Neurone? Rolle von Kalzium?	–
Disulfiram	250–500 mg/Tag	Inhibition der Acetaldehyddehydrogenase	–
Naltrexon	50 mg/Tag	µ‑Opioid-Rezeptor-Antagonist	Opioidabhängigkeit
Nalmefen	18 mg/Tag	µ- und δ‑Opioid-Rezeptor-Antagonist, partieller Agonist am κ‑Opioid-Rezeptor	–
Baclofen	3–80 mg/Tag (bis 270 mg)	GABA_B_-Rezeptor-Agonist (metabotrop)	Spastik
Gabapentin	900–1800 mg/Tag	Unklar, blockiert spannungsabhängige Kanäle. Keine Wirkung über GABA-Rezeptoren	Epilepsie, neuropathischer Schmerz
Ondansetron	0,5 mg/Tag	5‑HT_3_-Antagonist	Antiemetikum bei Krebs (Chemotherapie)
Prazosin/Doxazosin	Bis zu 16 mg/Tag	α1-Rezeptor-Agonist	–
Topiramat	Bis 300 mg/Tag	Nicht völlig klar, antikonvulsives Medikament, erhöht die GABA_A_-vermittelte neuronale Aktivität und antagonisiert AMPA- und Kainat-Glutamat-Rezeptoren, außerdem spannungsabhängige Kanäle, schwacher Inhibitor verschiedener anderer Enzyme	Epilepsie, Migräne, Lennox-Gastaut-Syndrom
Vareniclin	2 mg/Tag	Partieller Agonist am nikotinischen α4β2-Acetylcholinrezeptorsubtyp	Rauchen
γ‑Hydroxy-Buttersäure, GHB	–	Präkursor von GABA (schwacher Agonist am GABA_B_-Rezeptor) Glutamat, Glycin	Narkolepsie

Die neurobiologischen und neurochemischen Grundlagen der Alkoholabhängigkeit sind komplex, werden aber mittlerweile gut verstanden (Übersicht in [95]). Zentrale Strukturen bei der Wirkung von Rauschdrogen sind dopaminerge Neurone im mesolimbischen Bereich (ventrales Tegmentum, Nucleus accumbens) und ihre Projektion in den präfrontalen Kortex, der für Kontrollfunktionen und die Inhibition dysfunktionalen Verhaltens verantwortlich ist. Für Belohnung und Belohnungsantizipation spielt die Ausschüttung von Dopamin im mesolimbischen Bereich eine entscheidende Rolle. Ein abhängiger Konsum von Alkohol entwickelt sich aus einem Zusammenspiel von positiven (z. B. alkoholinduzierte Entspannung, Euphorie) und negativen Konsequenzen (z. B. Entzugssyndrome). Kurz zusammengefasst werden neurochemisch für die positiv verstärkenden Wirkungen vor allem Effekte auf Dopamin, das endogene Opioidsystem, das serotonerge und GABAerge sowie das endogene Cannabinoidsystem verantwortlich gemacht, für die negative Verstärkung vor allem die vermehrte Freisetzung von Kortikotropin-Releasing-Faktor, die GABAerge Down-Regulierung sowie Veränderungen im glutamatergen System (Übersicht in [105]). Aber auch appetitregulierende Hormone wie Ghrelin scheinen von Bedeutung zu sein [27]. Chronischer Alkoholkonsum führt zu erheblichen adaptiven Veränderungen und Anpassungen, vor allem der Rezeptorfunktionen im Gehirn, wobei es beim Alkoholentzug zu einer vermehrten Freisetzung exzitatorischer Neurotransmitter bei erhöhter Rezeptorempfindlichkeit und einer Übererregbarkeit des Gehirns kommt.

Bislang sind nur wenige Medikamente zur Alkoholentwöhnung zugelassen, obwohl mittlerweile eine ganze Reihe von Substanzen untersucht wurde (Übersicht in [23, 33, 53, 57]). Es gibt etablierte methodische Standards für die Durchführung von Pharmakotherapiestudien bei Alkoholabhängigkeit, wobei primäre Outcome- oder Responderkriterien entweder eine Verbesserung der Abstinenzrate, eine Erhöhung der Zeit bis zum ersten Konsum, eine Verminderung der Rückfallraten oder eine Trinkmengenreduktion sind (siehe [48, 88]). In verschiedenen Therapiestudien werden dabei sehr unterschiedliche Outcomekriterien verwendet [6, 25, 40, 76]. Sekundäre Outcomekriterien sind biologische Marker (Transaminasen, Carbohydrate-defizientes Transferrin [CDT], Ethylglukuronid), sozioökonomische Faktoren („health care utilization“) oder Wiederaufnahmen in Kliniken (Übersicht in [99]). Zuletzt wurde von einer Expertengruppe die WHO-Klassifikation unterschiedlicher Risikoniveaus („drinking risk levels“) als Outcomekriterium für Pharmakotherapiestudien empfohlen [26].

Voraussetzung für einen positiven Wirksamkeitsnachweis in klinischen Studien ist unter anderem, dass die pharmakologischen Interventionen auch ausreichend implementiert werden. Hohe Missing- und Drop-out-Raten, wie sie für den Bereich der Alkoholentwöhnung typisch sind, verhindern eine ausreichende Treatment-Implementierung und damit auch die Chancen, in Studien einen tatsächlich vorhandenen Effekt nachweisen zu können [88]. Wichtig ist zudem, dass die Auswahl der Outcomekriterien dem Wirkmechanismus der Substanz angepasst ist.

## Substanzen

### Disulfiram

Viele Jahrzehnte war Disulfiram das einzige zur Behandlung der Alkoholabhängigkeit zugelassene Medikament, welches wegen geringer Verschreibungszahlen in Deutschland nicht mehr vertrieben wird. Disulfiram beeinflusst nicht die biochemischen Effekte von Alkohol, sondern inhibiert das Enzym Aldehyddehydrogenase, welches das ansonsten schnell verstoffwechselte erste Abbauprodukt von Alkohol, Acetaldehyd, metabolisiert. Toxische erhöhte Acetaldehydspiegel führen zur sogenannten Disulfiram-Alkohol-Reaktion, einer künstlich herbeigeführten Vergiftung mit Übelkeit, Erbrechen, Kreislaufproblemen, Schweißausbrüchen, Hypertension, in schweren Fällen auch kardiovaskulären Reaktionen oder Kollaps (Übersicht in [70, 87]). Die therapeutische Wirkung von Disulfiram basiert primär auf dessen Unverträglichkeit mit Alkohol; dadurch nimmt die Substanz als Aversivtherapeutikum eine Sonderstellung unter den pharmakologischen Therapien der Alkoholabhängigkeit ein. Die klinische Wirkung von Disulfiram basiert auf der Antizipation dieser unerwünschten Effekte. Entscheidend für die therapeutische Wirkung scheint primär die gedankliche Vorwegnahme der Unverträglichkeit mit Alkohol zu sein, nicht die pharmakologische Wirkung der Substanz bzw. das tatsächliche Auftreten einer Alkohol-Disulfiram-Reaktion selbst.

Die größte zur Frage der Wirksamkeit von Disulfiram durchgeführte Untersuchung [30] konnte keinen Wirknachweis erbringen. Die Behandlungsergebnisse bei Supervision der Einnahme von Disulfiram scheinen der nichtsupervidierten Einnahme überlegen zu sein. Eine gute Compliance konnte im Rahmen des Projekts ALITA (Ambulante Langzeit-Intensivtherapie für Alkoholkranke) bei überwachter Einnahme nachgewiesen werden [49].

Wie eine Metaanalyse [92] mit Subgruppenanalyse unter Berücksichtigung des Studiendesigns deutlich macht, zeigt Disulfiram in offenen, nicht aber in randomisierten Doppelblindstudien positive Effekte. Dies ist auf den erwartungsvermittelten Wirkmechanismus der Substanz zurückzuführen: Indem die abstinenzunterstützenden Effekte von Disulfiram im Gegensatz zu anderen pharmakologischen Methoden der Rückfallprophylaxe nicht pharmakologisch, sondern ausschließlich durch Erwartung vermittelt sind, treten die erwartungsinduzierten Therapieeffekte in Studien mit verblindetem Design sowohl in der Interventions- als auch in der Kontrollgruppe auf. Dadurch ist ein Effektnachweis in randomisierten Doppelblindstudien für Disulfiram aus theoretischer Sicht gar nicht möglich [70, 87]. So zeigte sich in einer randomisierten Cross-over-Studie, in der die Erwartung, Disulfiram eingenommen zu haben, experimentell manipuliert wurde, auch bei placebobehandelten Probanden eine Abnahme der Reizreaktivität („cue reactivity“) auf alkoholassoziierte Reize [91]. Entgegen den methodischen Standards, wie wir sie aus der Wirksamkeitsprüfung anderer Substanzen kennen, sind die Aussagen über die Wirkeffekte zu Disulfiram aus Studien mit offenem Design zuverlässiger [87].

Trotz einer wieder zunehmenden Verordnung von Disulfiram hat die Herstellerfirma Nycomed im Jahr 2011 auf die Erneuerung der Zulassung verzichtet und die Produktion von Disulfiram eingestellt. Der Wirkstoff besitzt in Deutschland derzeit keine Zulassung, kann jedoch auf Basis patientenbezogener Anforderungen über internationale Apotheken bezogen werden. Der spezifische Wirkmechanismus von Disulfiram und die enge Verzahnung von therapeutischem Nutzen und gesundheitlicher Gefährdung erfordert eine besondere Sorgfaltspflicht bei der Information und Aufklärung des Patienten. So ist in jedem Fall sicherzustellen, dass sich der Patient der Notwendigkeit einer absoluten Abstinenz bewusst ist und eine hohe Abstinenzzuversicht unter Disulfiramtherapie signalisiert. Darüber hinaus sollte die Therapie mit Disulfiram der persönlichen Therapiepräferenz des Patienten entsprechen. Dessen Beteiligung an der Therapieplanung im Sinne einer partizipativen Entscheidungsfindung erweist sich vor dem Hintergrund des Wirkmechanismus von Disulfiram nicht nur im Hinblick auf die Compliance relevant, sondern determiniert darüber hinaus auch das individuelle Risiko einer Therapie mit Disulfiram.

### Acamprosat

Die Substanz ist jetzt seit über 20 Jahren klinisch verfügbar, ihr Wirkmechanismus aber immer noch nicht vollständig geklärt. Acamprosat interagiert nicht mit Alkohol oder anderen Psychopharmaka und hat auch keine psychotropen Effekte, die Substanz wird schlecht resorbiert, sodass bei einem Körpergewicht von über 60 kg die Einnahme von 6 × 330 mg (1998 mg) notwendig ist, was mitunter auch zu Compliance-Problemen beitragen kann. Für Acamprosat wird eine Wirkung über glutamaterge Neurone vermutet, auch wurde die Vermutung geäußert, wonach die Wirkung ausschließlich auf das Kalziumatom des Acamprosatmoleküls zurückführen ist [96].

Acamprosat wurde intensiv untersucht. Es gibt mehrere Metaanalysen ([23, 43, 57, 85]; vgl. Tab. 2). In den beiden größeren Metaanalysen wurden 27 Studien mit über 7000 Patienten eingeschlossen [43, 57]. Das Cochrane-Review zu Acamprosat [85] zeigt unter anderem eine signifikante Reduktion des Konsumrisikos auf 81 % des entsprechenden Risikos in der Kontrollgruppe (relatives Risiko [RR] = 0,86; 95 %-KI 0,81–0,91). Bei insgesamt heterogenen Behandlungsergebnissen wurde im Vergleich zu Placebo eine Reduktion der Trinktage gezeigt. Acamprosat ist in Europa von der European Medicines Agency und in den USA von der FDA zugelassen worden. Acamprosat wird üblicherweise gut toleriert, die häufigste milde Nebenwirkung von Acamprosat ist Durchfall (weicher Stuhl), der nach einigen Tagen häufig abklingt.

**Table 2:** 

Autor	Jahr	RCT (gesamt)	*N* (gesamt)	Konsum – Gesamteffekt	Konsum – KI	Rückfall – Gesamteffekt	Rückfall – KI
Rösner et al. [85]	2010	24	6915	0,86 (RR)	0,81 bis 0,91	0,99 (RR)	0,94 bis 1,04
Maisel et al. [57]	2013	16	4349	0,36 (Hedges g)	0,25 bis 0,47	0,07 (Hedges g)	−0,08 bis −0,22
Jonas et al. [43]	2014	16	4847	−0,09 (RD)	−0,14 bis −0,04	−0,01 (RD)	−0,04 bis −0,03
Donoghue et al. [23]	2015	22	5236	0,83 (RR)	0,78 bis 0,89	–	–

*RCT* „Randomized controlled trial“, *N* Stichprobenumfang, *KI* Konfidenzintervall, *RR* relatives Risiko, *RD* Risikodifferenz

Es gibt zahlreiche placebokontrollierte Doppelblindstudien mit Acamprosat, wenige Vergleichsstudien zu Naltrexon, wobei die größte Untersuchung, die in den USA durchgeführte COMBINE-Studie [9], keinen Wirknachweis von Acamprosat erbrachte. Auch die deutsche Studie [46] zeigte bessere Behandlungsergebnisse für den Opiatantagonisten Naltrexon als für Acamprosat bei gleichzeitigem Vorteil einer kombinierten Behandlung unter Nutzung beider Substanzen.

### Opiatantagonisten (Naltrexon, Nalmefen)

Naltrexon ist ein nichtselektiver Opioidantagonist am µ‑, κ‑ und δ‑Opioidrezeptor. Zahlreiche pharmakologische Untersuchungen haben gezeigt, dass Naltrexon die subjektiven Effekte von Alkohol vermindern kann, z. B. Alkoholverlangen (Craving), alkoholinduzierte Stimulation, Sedation und auch negative Stimmungen nach Alkoholkonsum [80]. Naltrexon blockiert die positiv verstärkenden „hedonischen“ Effekte von Alkohol, darüber sind über die Beeinflussung des mesolimbischen Dopaminsystems auch motivationale Effekte anzunehmen. Die Interaktionen zwischen Opioidrezeptorblockade und Dopaminausschüttung sind komplex und dynamisch, gesichert ist aber, dass durch die Blockade des Opioidendorphinsystems indirekt auch die Ausschüttung von Dopamin im Nucleus accumbens reguliert wird (Übersicht in [37]).

In Untersuchungen wurde üblicherweise eine Dosis von 50 mg (1 Tbl. oral) untersucht. Die ersten Untersuchungen wurden von O’Malley et al. [73] und Volpicelli et al. [104] durchgeführt, zahlreiche andere placebokontrollierte Doppelblindstudien haben sich angeschlossen. Metaanalysen haben gezeigt, dass Naltrexon vor allem die Trinkmenge bzw. die Rückfallrate zu schwerem Trinken vermindert ([23, 43, 57, 86]; vgl. Tab. 3) und weniger Einfluss auf die Abstinenzrate hat. Das Cochrane-Review zu Naltrexon [86] zeigt eine signifikante Reduktion des Risikos für einen Rückfall (= Konsum von 5 „standard drink units“ oder mehr) auf 83 % des Risikos in der Kontrollgruppe (RR = 0,83; 95 %-KI 0,76–0,90). Eine Depotform von Naltrexon ist in den USA, nicht aber in Deutschland zur Behandlung der Alkoholabhängigkeit zugelassen [32].

**Table 3:** 

Autor	Jahr	RCT (gesamt)	*N* (gesamt)	Konsum – Gesamteffekt	Konsum – KI	Rückfall – Gesamteffekt	Rückfall – KI
Rösner et al.	2010	50	7793	0,96 (RR)	0,92 bis 1,00	0,83 (RR)	0,76 bis 0,90
Maisel et al. [57]	2013	45	5434	0,12 (Hedges g)	0,05 bis 0,18	0,19 (Hedges g)	0,12 bis 0,25
Jonas et al. [43]	2014	19	2875	−0,05 (RD)	−0,10 bis −0,002	−0,09 (RD)	−0,13 bis −0,04
Donoghue et al. [23]	2015	27	4199	0,92 (RR)	0,86 bis 1,00	0,85 (RR)	0,78 bis 0,93

*RCT* „Randomized controlled trial“, *N* Stichprobenumfang, *KI* Konfidenzintervall, *RR* relatives Risiko, *RD* Risikodifferenz

Basierend auf dem Datensatz der Cochrane-Analyse [86] wurde vor kurzem eine Metaanalyse über 38 Studien mit 11.194 Teilnehmern publiziert [13], die eine Reihe von Nebenwirkungen identifizierte, die bei Naltrexon häufiger sind als bei Placebo (verminderter Appetit, Schläfrigkeit, Übelkeit, Benommenheit, Schwitzen und Erbrechen). Bei Opiatabhängigkeit führt die Gabe von Naltrexon zur Auslösung eines Opioidentzugssyndroms.

**Figure 1:**
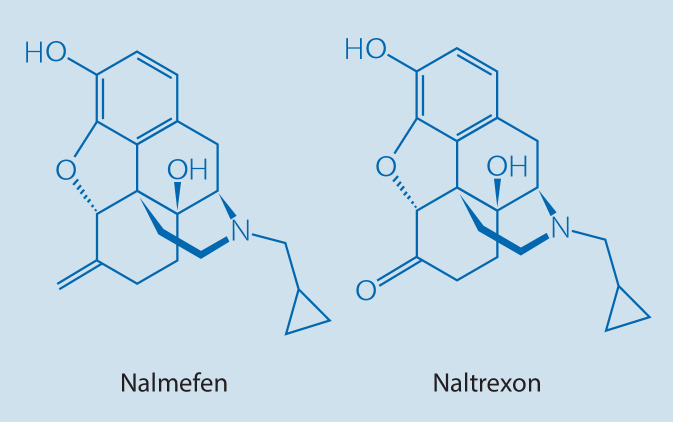

Nalmefen hat bis auf ein Atom eine ähnliche Struktur wie Naltrexon, aber ein etwas anderes Rezeptorprofil (Abb. 1). Nalmefen ist wie Naltrexon ein Antagonist am µ‑Opioidrezeptor und Modulator am δ‑Opioidrezeptorsubtyp. Die Wirkung von Nalmefen wird darüber hinaus über den κ‑Rezeptor vermittelt, wo sich partiell agonistische und antagonistische Wirkungen zeigen [94].

Basierend auf einer Reihe von kleineren Studien wurde Nalmefen in den vergangenen Jahren ausschließlich zur Trinkmengenreduktion eingesetzt. In den letzten Jahren wurde die Wirksamkeit von Nalmefen zur Trinkmengenreduktion in drei größeren placebokontrollierten Untersuchungen zur Trinkmengenreduktion unter Verwendung eines „As-needed“-Ansatzes geprüft [35, 60, 100, 101]. Dabei wird Nalmefen bei Bedarf, also zum Beispiel in Situationen eines erhöhten Cravings oder anderer Risikofaktoren, eingesetzt. Die Untersuchungen zeigen insgesamt eine mäßige Reduktion der Trinkmenge. Allerdings ist zu berücksichtigen, dass in die Studien nur Patienten mit leichterem und moderatem Schweregrad einer Alkoholabhängigkeit eingeschlossen wurden.

2013 wurde die Substanz von der European Medicines Agency zugelassen, in den USA ist Nalmefen nicht verfügbar. Auch wenn Harm-reduction-Strategien bei Alkoholabhängigkeit prinzipiell als sinnvoll zu bewerten sind [28, 72, 98], müssen die Ergebnisse auch vor dem Hintergrund methodischer Aspekte interpretiert werden. So ergeben sich in einer Metaanalyse mit 5 randomisierten, kontrollierten Studien zu Nalmefen [75] Hinweise auf selektive Drop-out-Raten sowie eine mangelnde Repräsentativität der Stichprobe. So liegt die durchschnittliche Trinkmenge der Probanden unter den üblichen Durchschnittswerten alkoholabhängiger Samples.

Positive Ergebnisse wurden aber vor Kurzem von einer weiteren Arbeitsgruppe publiziert [66], die Nalmefen 10 und 20 mg gegen Placebo in einer großen Studie über 24 Wochen bei alkoholabhängigen Patienten untersuchte und dabei erneut eine Reduktion des „heavy drinking“ im Vergleich zu Placebo fand. Auch offene Studien mit Nalmefen haben einen positiven Effekt gezeigt [16]. Der „As-needed“-Ansatz, vor allem bei weniger schwer betroffenen Patienten mit Alkoholkonsumstörungen, ist ein interessanter, den es weiter zu untersuchen gilt.

Eine Reihe von anderen Substanzen habt ein Potenzial im Hinblick auf die pharmakologische Rückfallprophylaxe bei Alkoholabhängigkeit, wobei die wichtigsten Medikamente aus anderen Indikationsbereichen stammen und die neurochemischen Effekte extrem unterschiedlich sind – was unterstreicht, dass die neurobiologische Alkoholforschung bislang keine „magic bullets“ entwickelt hat (Tab. 1).

### Baclofen

Baclofen ist ein selektiver GABA_B_-Rezeptor-Agonist, der als Arzneistoff aus der Gruppe der Muskelrelaxanzien zur Behandlung von Spastizität und bei multipler Sklerose eingesetzt wird. Das Interesse ist nach einem Eigenbericht eines mittlerweile verstorbenen französischen Arztes, der seine „Heilung“ vom Alkoholismus auf eine Hochdosistherapie mit Baclofen bis 250 mg/Tag zurückführte [7], gestiegen. Baclofen wird rasch resorbiert.

Eine Reihe von randomisierten Therapiestudien mit verschiedenen Dosierungen und auch unterschiedlichen Ergebnissen wurde durchgeführt [1–3, 31, 67]. Die Ergebnisse der US-amerikanischen Studie [31], der australischen [67], der holländischen [11] und der israelischen Studien [78] konnten keine Überlegenheit gegenüber Placebo nachweisen. In der BacALD-Studie war Baclofen ein wirksames Medikament, höhere Dosen waren mit schwereren Nebenwirkungen assoziiert [68]. Eine deutsche Untersuchung [69] mit Baclofen bis 270 mg/Tag zeigte deutlich positive Effekte auf die Abstinenzraten.

Baclofen ist in Frankreich zugelassen, auch wenn das Sicherheitsprofil der Substanz kritisch diskutiert wird [84, 90]. Nebenwirkungen sind Müdigkeit, Schlafstörungen, Übelkeit und Benommenheit. Vor allem die Hochdosisgabe erscheint problematisch [17] und ist für eine höhere Rate von Hospitalisierungen und Todesfällen verantwortlich. Aufgrund alkoholähnlicher Wirkungen von Baclofen im Gehirn wird diskutiert, inwieweit die Therapie mit Baclofen als Substitutionsbehandlung bei Alkoholismus bezeichnet werden kann [20]. In Frankreich wurden zwei sehr große Untersuchungen durchgeführt. In der ALPADIR-Studie [82] konnte keine Überlegenheit von Baclofen gegenüber Placebo nachgewiesen werden, in der BACLOVILLE-Studie [41] zeigte sich ein signifikanter Effekt in der Baclofengruppe für Abstinenz und die Reduktion der WHO-Risikoniveaus.

In einer Metaanalyse mit 14 randomisierten klinischen Studien wurden keine signifikanten Effekte von Baclofen nachgewiesen, dies ebenso in einer Post-hoc-Analyse der Studien mit höheren Dosen [15]. Eine aktuelle Cochrane-Analyse [65] mit insgesamt 12 randomisierten klinischen Studien konnte die Wirksamkeit von Baclofen ebenfalls nicht bestätigen. Allerdings wurde auch eine starke Heterogenität der Effektstärken deutlich, die für den Einfluss von Stichproben und Designmerkmalen spricht. Die in einigen Studien mit hoher Studienqualität nachgewiesenen positiven Effekte (z. B. [69]) sprechen weiter für eine mögliche Wirksamkeit von Baclofen. In weiteren Studien ist zu prüfen, inwieweit bestimmte Merkmale der Behandlung (z. B. individuelle Dosisanpassung) die Wirksamkeit von Baclofen beeinflussen.

### Antikonvulsiva (Gabapentin, Pregabalin, Topiramat)

Gabapentin ist als Antiepileptikum und zur Behandlung der Neuralgie und des Restless-legs-Syndroms zugelassen (Dosierungen 300 bis 1800 mg/Tag). Die American Psychiatric Association (APA) empfiehlt Gabapentin und das unten besprochene Topiramat bei Unverträglichkeit von Acamprosat und Naltrexon oder bei fehlendem therapeutischem Ansprechen auf die gängigen Anti-Craving-Medikamente. Der Wirkmechanismus von Gabapentin ist nicht völlig klar, es beeinflusst spannungsabhängige Ca-Kanäle [62] und verstärkt die GABAerge Aktivität, bindet aber nicht an GABA-Rezeptoren.

Eine kürzlich erschienene Metaanalyse [52] mit 7 randomisierten, kontrollierten Therapiestudien zu Gabapentin fand einen mittelgradigen Effekt auf die Anzahl der „heavy drinking days“, alle anderen Outcomekriterien waren nicht signifikant. Eine weitere Metaanalyse [5] konnte auf der Grundlage von 10 Studien einen moderaten Effekt auf die Symptome eines Alkoholentzugs und auf das Alkoholcraving nachweisen. Pharmacovigilance-Meldungen des FDA Adverse Events Reporting System finden Hinweise auf ein Missbrauchspotenzial der Substanz [103], das vermutlich auf den GABAergen Wirkmechanismus von Gabapentin und seine entspannenden Effekte zurückzuführen ist.

Für Pregabalin liegen nur einige wenige Befunde für die Behandlung des Alkoholentzugssyndroms vor [29], nichts zur Rückfallprophylaxe. Ohnehin wäre diese Substanz wegen ihres Suchtpotenzials als Anti-Craving-Medikament kritisch zu bewerten.

Topiramat ist ein Antiepileptikum, dass am GABA-Rezeptor wirkt und gleichzeitig auch die Aktivität glutamaterger Rezeptorsubtypen reduziert [50, 51]. Es gibt eine Reihe randomisierter Studien zur Wirksamkeit von Topiramat, meist wurden Dosen von 200 bis 300 mg eingesetzt. Wie Metaanalysen zeigen, sind die therapeutischen Effekte mit denen etablierter Anti-Craving-Substanzen vergleichbar, die Datenlage ist jedoch nicht ausreichend, um aussagekräftige Schlussfolgerungen abzuleiten (z. B. [12, 77]). Als häufigste Nebenwirkung zeigen sich unter anderem Schwindel, Müdigkeit, Parästhesien und Appetitverlust, welche hinsichtlich Verträglichkeit und Compliance problematisch sind.

### Ketamin

Zu den interessanten neuen Ansätzen gehört der nichtkompetitive glutamaterge NMDA-Rezeptor-Antagonist Ketamin [18]. Hier konnte experimentell gezeigt werden, dass Ketamin verstärkende Effekte von Alkohol vermindern kann [19]. Kontrollierte klinische Studien stehen noch aus.

### Vareniclin

Vareniclin ist ein partieller Agonist am α4β2-Nikotin-Rezeptor und ein voller Agonist an nikotinischen Acetylcholinrezeptoren (nAChR) und für die Behandlung von Nikotinabhängigkeit zugelassen. Es gibt auch einige wenige Untersuchungen zur Wirksamkeit bei alkoholabhängigen Patienten [54, 56], darunter auch Untersuchungen mit negativem Ergebnis [21]. Der Einsatz bei komorbiden Rauchern ist naheliegend. Aktuell laufen einige Studien, auch Vergleichsuntersuchungen zu Naltrexon [54]. Eine aktuelle Metaanalyse mit über 9 placebokontrollierten Doppelblindstudien (*N* = 585, Behandlungsdauer 4–13 Wochen) zeigt, dass Vareniclin im Vergleich zu Placebo nicht die Zahl der „heavy drinking days“, aber die Menge des konsumierten Alkohols insgesamt vermindern konnte [74].

### γ-Hydroxybutyrat (GHB)

Kontrovers wird der Einsatz von „sodium oxybate“ (SMO) und γ‑Hydroxybutyrat (GHB), einer über den GABA-Rezeptor wirkenden stark psychotropen Substanz, bewertet, die in Österreich und in Italien zur Behandlung von Alkoholentzugssyndromen und Alkoholabhängigkeit zugelassen ist (Übersicht in [44]). GHB hat selber ein starkes Suchtpotenzial und traurige Berühmtheit als „rape drug“ erlangt, als Substanz, die im Rahmen von Straftaten genutzt wird und Amnesie hervorruft, sodass sich die Opfer nicht mehr an die Tat oder den Tathergang erinnern können. Eine Metaanalyse [14] zeigt unter Einschluss von 7 randomisierten, kontrollierten Studien eine signifikante Überlegenheit von GHB gegenüber Placebo für verschiedene Outcomes der Alkoholentwöhnung, im niedrigen Dosisbereich scheint die Substanz zudem gut verträglich zu sein [44]. Dem Wirksamkeitsnachweis steht das vergleichsweise hohe Missbrauchspotenzial von GHB gegenüber, welches die Eignung der Substanz als pharmakologische Unterstützung der Alkoholentwöhnung stark einschränkt. Zusätzlich problematisch für die Verwendung der Substanz im Bereich der Alkoholtherapie ist die potenzierende Wirkung von GHB auf Alkohol (Übersicht in [102]).

### Antidepressiva und Antipsychotika

Konventionelle psychotrope Substanzen wie z. B. Antidepressiva oder Antipsychotika, die das Dopaminsystem blockieren [47], haben sich in der pharmakologischen Rückfallprophylaxe zumindest bei nicht psychisch kranken Alkoholabhängigen als nicht wirksam erwiesen (Übersicht in [93]).

### Weitere Substanzen

Andere Untersuchungen, bei denen verschiedene Substanzen eingesetzt worden sind, umfassen Ondansetron [42, 45], den α1-Rezeptor-Blocker Prazosin und Doxazin [36], den Glukuronidrezeptorblocker Mifepriston, außerdem Oxytozin und weitere Substanzen [105]. Für Prazosin liegen einige Untersuchungen vor [54], die letzte von Simpson et al. [89], die 92 Patienten mit 16 mg Prazosin oder Placebo behandelte und einen gewissen klinischen Effekt auf die Trinkmenge beschreiben konnte. Als Nebenwirkung tritt Benommenheit relativ häufig auf.

## Pharmakogenetik

Hier liegen bislang nur relativ wenig neue Befunde vor (Übersicht in [38, 39, 79, 83]), was angesichts der bislang begrenzten Auswahl pharmakotherapeutischer Optionen nicht überrascht. Am ehesten auch von klinischem Interesse waren bislang funktionelle Polymorphismen im OPRM1- und OPRK1-Gen, die wahrscheinlich die Effekte von Opiatantagonisten wie Naltrexon modifizieren. Dazu liegt inzwischen eine Reihe experimenteller und klinischer Befunde vor [79, 83]. Eine aktuelle Metaanalyse mit 7 randomisierten, kontrollierten Studien fand einen moderaten Effekt des Asn40Asp-SNP für das Outcome „drinks per day“, ansonsten keine weiteren Hinweise, dass das rs1799971-G-Allel im OPRM1-Gen einen Einfluss auf das Ansprechen auf eine Therapie mit Naltrexon hat [39]. Für Acamprosat könnten Variationen in den glutamatergen Rezeptoren (GATA4, GREN2b) von Bedeutung sein, für Topiramat Polymorphismen in den glutamatergen AMPA- und Kainatrezeptoren (GRIK1 und 2).

Eine Übersichtsarbeit über den Einfluss serotonerger Genvariationen auf das Ansprechen pharmakotherapeutischer Methoden der Rückfallprophylaxe bei Abhängigkeitserkrankungen weist auf die Bedeutung von Genen für das Enzym Tryptophanhydroxylase 2 (*TPH2*) und den Serotonintransporter (*SLC6A4*) hin, welche die Wirkung von Ondansetron und Disulfiram modulieren [10].

In der BacALD-Studie wurde für Baclofen die Relevanz des RS29220-SNP im GABA_B_-Rezeptor für den Therapieerfolg und möglicherweise auch für Unverträglichkeitsreaktionen für Baclofen gezeigt [68].

## Schlussfolgerung

Bislang sind nur wenige Medikamente zur Pharmakotherapie der Alkoholabhängigkeit zugelassen, einige andere Substanzen, die überwiegend aus anderen medizinischen Indikationsbereichen stammen, wie Baclofen, Topiramat, Vareniclin oder Gabapentin, könnten künftig eine größere Rolle spielen. Die neurochemischen Effekte und Grundlagen der einzelnen Substanzen unterscheiden sich erheblich. Lückenhaft sind die klinischen Daten insbesondere auch bezüglich der Wirksamkeit von Anti-Craving-Substanzen bei komorbider psychischer Störung.

Klinisch von Bedeutung ist aber, dass bereits die bislang verfügbaren Substanzen kaum eingesetzt werden, stattdessen werden häufig konventionelle Psychopharmaka (ohne Wirkungsnachweis in diesem Bereich) eingesetzt, namentlich Antidepressiva, obwohl sie zur Trinkmengenreduktion unwirksam sind [4]. Somit wird eine bereits verfügbare pharmakotherapeutische Option zur Rückfallprophylaxe bei Alkoholabhängigen nicht genützt, man könnte auch sagen „verschenkt“. Stellt man die Verordnungszahlen in Relation zu der hohen Zahl der von Alkoholabhängigkeit betroffenen Personen, kann hier von einer deutlichen Unterversorgung abhängiger Patienten mit pharmakologischen Interventionen gesprochen werden [97]. Diese sind natürlich stets als adjuvante Strategie zur Begleitung und Ergänzung psychotherapeutischer Methoden zu verstehen.

Die immer noch zögerliche Verordnung von Anti-Craving-Substanzen ist durch die Datenlage kaum begründbar. So werden mit Anti-Craving-Substanzen Effekte erzielt, die in ihrer Ausprägung durchaus mit etablierten Therapien anderer psychiatrischer und somatischer Behandlungsbereiche vergleichbar sind (vgl. z. B. [55]). Hinzu kommt, dass die tatsächlichen Effekte der Anti-Craving-Behandlung aufgrund methodischer Besonderheiten im Bereich der Abhängigkeitstherapie durch klinische Studien eher unterschätzt als überschätzt werden dürften [88].

An der zurückhaltenden therapeutischen Nutzung von Anti-Craving-Substanzen dürften vermutlich auch psychologische Faktoren wie eine geringe Erfolgserwartung oder die Befürchtung einer eventuellen „Suchtverlagerung“ beteiligt sein. Die Prüfung eigener Vorbehalte, das offene Ansprechen etwaiger Bedenken des Patienten und dessen Beteiligung an einer gemeinsamen Entscheidungsfindung sind im Bereich der Anti-Craving-Behandlung von besonderer Bedeutung.

Auch wenn sich die Basis der klinisch wirksamen Substanzen nur zaghaft zu verbreitern scheint, kann eine zunehmende Anpassung der Strategien an die individuellen Bedürfnisse und Präferenzen des Patienten eine Zunahme der Compliance erzielen. Damit sollte auch das klinische und wissenschaftliche Interesse an den Substanzen wieder stärker in den Fokus rücken.
